# Transcutaneous electrical acupoint stimulation improves pulmonary function by regulating oxidative stress during one-lung ventilation in patients with lung cancer undergoing thoracoscopic surgery: a randomized controlled trial

**DOI:** 10.1186/s12906-023-04304-1

**Published:** 2023-12-16

**Authors:** Songxu Ju, Meinv Liu, Bei Wang, Dongdong Yu, Huanhuan Zhang, Meng Zhang, Jianli Li

**Affiliations:** 1 Department of Anesthesiology, Hebei General Hospital, 348 Heping West Road, Shijiazhuang, 050051 Hebei China; 2 Department of Gynecology, Hebei General Hospital, Shijiazhuang, China

**Keywords:** Transcutaneous electrical acupoint stimulation, One-lung ventilation, Oxidative stress, Pulmonary function

## Abstract

**Background:**

Our aim was to evaluate the efficacy of transcutaneous electrical acupoint stimulation (TEAS) on oxidative stress induced by one-lung ventilation, lung function, and postoperative quality of recovery in patients with lung cancer.

**Methods:**

The participants (n = 80) were assigned to the sham group and TEAS group. TEAS on bilateral Feishu (BL13), Zusanli (ST36), and Hegu (L14) was performed 30 minutes before induction of anesthesia and continued until the end of the surgery. In the sham group, the same acupoints were selected without electrical stimulation. PaO2/FiO2, intrapulmonary shunt ratio (Qs/Qt), alveolar-arterial oxygen tension (A-aDO2), and respiratory index (RI) were calculated to evaluate lung function before one-lung ventilation (T0), 30 min after one-lung ventilation (T1), 1 h after one-lung ventilation (T2), and 10 min after resuming two-lung ventilation (T3). The levels of malondialdehyde (MDA) and superoxide dismutase (SOD) were detected to estimate oxidative stress at T0, T1, T2, and T3. Secondary outcomes included removal time of thoracic drainage tube, duration of intensive care unit (ICU) stay, length of postoperative hospitalization, the incidence of postoperative pulmonary complications, and the Quality of Recovery-15 (QoR-15) score on postoperative day 1 and 2.

**Results:**

TEAS significantly increased PaO2/FiO2 at T1 and T2, while Qs/Qt, A-aDO2, and RI decreased remarkably from T1 to T3 (*P* < 0.05). Meanwhile, TEAS obviously decreased MDA and increased SOD activity at T2 and T3 (*P* < 0.05). Furthermore, TEAS also markedly shortened the length of ICU stay and hospital stay after surgery, whereas the QoR-15 score on postoperative day 1 and 2 was significantly higher (*P* < 0.05).

**Conclusions:**

TEAS could reduce oxidative lung injury during one-lung ventilation, thereby protecting pulmonary function and effectively accelerating the early recovery of patients with lung cancer.

**Trial registration:**

Chinese Clinical Trial Registry (ChiCTR2000038243).

## Introduction

One-lung ventilation (OLV) has allowed increasingly complex thoracic surgery and is a de facto indispensability with the increased use of minimally invasive techniques. Nevertheless, as a kind of nonphysiological ventilation mode, OLV has been viewed as a risk factor for acute lung injury (ALI) and is known to be associated with postoperative pulmonary complications [1]. Moreover, the nondependent lung is reventilated and reoxygenated after the restoration of two-lung ventilation, which leads to hypoxia-reoxygenation injury similar to ischemia-reperfusion injury with an increase in malondialdehyde (MDA) levels and reactive oxygen species (ROS) production [2]. The degree of oxidative stress is directly related to OLV duration and negatively correlated with arterial blood PaO_2_/FiO_2_ [3]. Protracted (> 1 h) OLV has been considered to be a potential cause of cardiovascular complications through the generation of oxidative stress [4], which also plays an essential part in the development of ALI and acute respiratory distress syndrome (ARDS) [5].

Acupuncture therapy is the treasure of Chinese medicine, which has been widely employed in China, Japan and South Korea. Currently, acupuncture therapy mainly includes acupuncture, electroacupuncture, and transcutaneous electrical acupoint stimulation (TEAS). Previous study showed that acupuncture could protect against myocardial ischemia-reperfusion injury through inhibiting oxidative stress in rats [6]. Besides, researches suggested that electroacupuncture could attenuate cerebral ischemia and reperfusion injury in rats via modulation of inflammation and oxidative stress [7, 8]. Moreover, many experimental studies showed a protective effect of electroacupuncture to alleviate lung injury induced by limb ischemia-reperfusion [9–11]. TEAS is a noninvasive intervention method that combines the effect of traditional acupuncture and transcutaneous electrical nerve stimulation (TENS). It had been shown to be a feasible method for sedative and postoperative analgesic [12], which can effectively reduce the levels of inflammatory factors and decrease the length of the hospital stay [13]. Nevertheless, it is unclear whether TEAS can improve lung function and the quality of postoperative recovery through regulating oxidative stress during one-lung ventilation.

Therefore, we conducted this study to explore the effect of TEAS on oxidative lung injury during one-lung ventilation, lung function, and postoperative quality of recovery in patients with lung cancer undergoing thoracoscopic surgery.

## Methods

### Study design

This is a single-blind, randomized controlled clinical trial with two parallel groups. Ethical approval was obtained from the Ethics Committee for Clinical Trial of Hebei General Hospital, China (ethics approval no. 2019-48, ethics approval date: 29/04/2019). Informed consent was signed by each participant (or legal representative). The clinical trial registration number was ChiCTR-2,000,038,243 (First trial registration: 15/09/2020).

### Sample size

G*Power software was used to calculate the sample size, which was determined based on the level of PaO_2_/FiO_2_ in our pilot study. Considering M1 = 169.25, M2 = 137.15, SD1 = 45.27, SD2 = 38.04, α = 0.05, Power = 80%, sample size was 28 in each group. Furthermore, assuming a dropout of 20% of cases, at least 34 patients were needed for each group. Consequently, 85 patients were finally included in our study.

### Participants

**Fig. 1:**
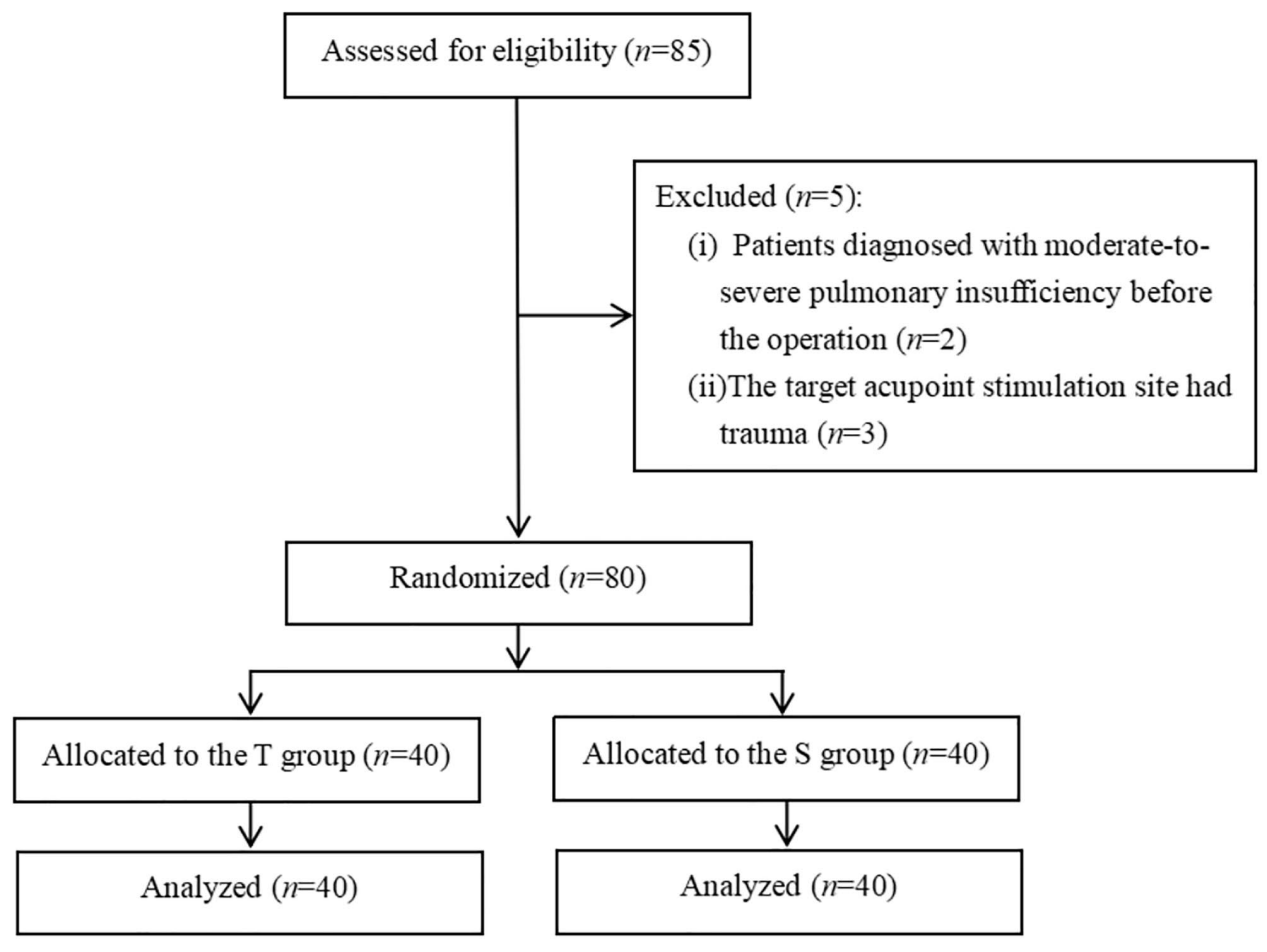
Study flow diagram of participants in the randomized trial

Eighty-five patients with lung cancer were recruited for thoracoscopic pulmonary lobectomy in the present study (Fig. 1). All patients were American Society of Anesthesiologists (ASA) II-III, aged 30–65 years, with adequate language skills and cognitive function. Exclusion criteria include: (1) patients who were diagnosed with moderate to severe pulmonary insufficiency preoperatively; (2) patients whose forced expiratory volume in 1 s (FEV_1_) < 50% or temperature > 37.5ºC and antioxidant agents intake; (3) patients who fitted with a pacemaker; (4) patients who had received acupoint stimulation or acupuncture; (5) patients whose stimulation site was infected or injured.

### Randomization and blinding

A trained researcher (B.W) allocated the participants the TEAS group or sham group in a 1:1 allocation according to a computer-generated random sequence. M.Z enrolled participants, and another two researchers (M.N.L and J.L.L) assigned participants to interventions. Concealed allocation was attained using sequentially numbered, opaque, and sealed envelopes. The attending anesthesiologists, surgeons, data collectors and the patients were unaware of the allocation. The participants were informed that they may feel various sensations, such as strong or mild tickling, or nothing during the TEAS procedure. They were told that just one parameter of TEAS was tested, so it was impossible for the participants to distinguish whether placebo or real TEAS [14].

### Anesthesia and surgery

All the patients were fasted for 6 h and prohibited to drink 2 h before the operation. Routine monitoring was established on all patients including electrocardiogram, peripheral capillary oxygen saturation (SpO_2_), bispectral index (BIS), heart rate (HR) and mean arterial pressure (MAP). Under local anesthesia, ultrasound-guided puncture and catheterization were performed on the radial artery for continuous blood pressure monitoring and arterial blood gas analysis.

Before induction, all patients were preoxygenated with 100% oxygen for at least 3 min. Induction of anesthesia was started with intravenous sufentanil 0.3 µg/kg, midazolam 0.05 mg/kg, etomidate 0.3 mg/kg, and rocuronium 1.0 mg/kg. After induction, a suitable size Double-Lumen Tube (DLT) was inserted into the left or right bronchus. The position of DLT was confirmed by auscultation and fiberoptic bronchoscope. Mechanical ventilation was used in a pressure-controlled ventilation-volume guaranteed (PCV-VG) mode for protective ventilation in this study. The ventilator parameters were as follow: tidal volume (V_T_) of 6-8mL/kg, respiratory frequency of 12 to 15/minute, inspiratory: expiratory (I: E) ratio of 1:2, fraction of inspired oxygen (FiO_2_) of 100%. During one-lung ventilation, the ventilator parameters were V_T_ of 5-6mL/kg, FiO_2_ of 80%, positive end-expiratory pressure (PEEP) of 5 cmH_2_O, and end-tidal carbon dioxide partial pressure was maintained from 35 to 45 mmHg. If SaO_2_ decreased to < 92%, FiO_2_ was increased temporarily to 100%, the position of DLT was verified, and recruitment maneuvers were performed. During maintenance of anesthesia, all patients were given sevoflurane, remifentanil, and rocuronium to maintain the BIS between 40 and 60. After the surgical intervention, the operated lung was reinflated with a positive pressure of 20 to 25 cmH_2_O to re-expand. If the MAP was less than 30% of baseline, the patients were administered with 6 mg hydrochloride ephedrine. Urapidil hydrochloride (0.10–0.15 mg/kg) was used if the MAP was > 120%. If the heart rate decreased to less than 50 beats per minute, 0.2 mg of atropine sulfate was administered intravenously. All anesthetic drugs were discontinued at the end of the operation. Perioperative fluids were administrated in a standardized way. After surgery, patients were transferred to the postanesthesia care unit (PACU) for routine postoperative care. Then, the anesthesiologist escorts the patients back to the thoracic surgery intensive care unit (ICU) for treatment.

### Intervention

Before anesthesia induction, patients in the TEAS group received TEAS treatment for 30 min at bilateral Feishu (BL13, located under the spinous process of the third thoracic vertebrae approximately 5 cm apart), Zusanli (ST36, located 5 mm below and lateral to the anterior tubercle of the tibia), and Hegu (L14, between the first and second metacarpal bones on the dorsum of the hand, the midpoint of the radial side of the second metacarpal bone) with a stimulator (SDZ-II model, Suzhou Medical Appliances Co. Ltd, Suzhou, China), which continued until the end of the operation.

These acupoints are based on traditional anatomical positions. Prior to each treatment, calibration was performed with the TEAS device. Electrode tabs were placed on the bilateral BL13, ST36, and L14. The TEAS was maintained at a frequency of 2/100 Hz using a dilatational wave throughout the procedure. The current intensity varied from 5 to 15 mA. Patients in the sham group were only connected to the electrodes without initiating electrical stimulation. All participants were blinded to the allocation. Both types of interventions were performed by a single investigator. The data were collected by another investigator who unaware of the distribution.

### Measurements

The primary outcome was PaO2/FiO2 in our study, the secondary endpoints included (1) variables of lung function and oxidative stress: Qs/Qt, respiratory index (RI), A-aDO2, MDA and SOD; (2) other relevant clinical parameters: MAP, HR, Hb, pH, PaCO2, removal time of thoracic drainage tube, duration of ICU stay, length of postoperative hospitalization, the Quality of Recovery-15 (QoR-15) score [15], and the incidence of postoperative pulmonary complications. Definitions for complications included pleural effusion and atelectasis [16].

Blood gas analysis was performed in all patients before one-lung ventilation (T0), 30 min after one-lung ventilation (T1), 1 h after one-lung ventilation (T2), and 10 min after resuming two-lung ventilation (T3) to calculate PaO_2_/FiO_2_, A-aDO_2_, RI, and intrapulmonary shunt ratio (Qs/Qt). Internal jugular vein samples were collected at T0, T1, T2, and T3 to detect SOD activity by xanthine oxidase method, while thiobarbituric acid was used as the substrate to detect MDA (Nanjing Ji Sheng Medical Technology Co, Ltd, Nanjing, China) according to previous studies [17, 18].

The following equations were used to calculate these parameters:

1 $$\begin{array}{l}{{\rm{Q}}_{\rm{S}}}{\rm{/}}{{\rm{Q}}_{\rm{t}}}{\rm{ = }}({\rm{PA - aD}}{{\rm{O}}_{\rm{2}}} \times {\rm{0}}.{\rm{0031}}) \div \\\,\,\,\,\,\,\,\,\,\,\,\,\,\,\,\,({\rm{PA - aD}}{{\rm{O}}_{\rm{2}}} \times {\rm{0}}.{\rm{0031 + 5}}),\end{array}$$

whereby PA-aDO_2_ = [FiO_2_ × (P_B_-P_H2O_)]-(PaCO_2_/*R*)-PaO_2_,

2 $${\rm{RI = PA - aD}}{{\rm{O}}_{\rm{2}}}{\rm{/Pa}}{{\rm{O}}_{\rm{2}}}$$

PA-aDO_2_ is the alveolar-arterial oxygen difference; PB is the barometric pressure (760 mmHg); P_H2O_ is the vapor pressure of water (47 mmHg); R is the respiratory quotient (0.8).

### Statistical analysis

Statistical analyses were conducted using SPSS 26.0 (IBM, Armonk, NY, USA). Data were presented as the mean ± standard deviations for continuous variables and as proportions for categorical variables. Normally distributed continuous data (determined by the Kolmogorov-Smirnov method) were compared using Student’s *t*-test. The Chi-square test was used to analyze categorical variables. Repeated measures analysis of variance (RM-ANOVA) were employed to analyze differences between different time points for the same indicator. A 2-tailed 𝑃 value less than 0.05 was regarded statistically different.

## Results

### Demographic and intraoperative characteristics of patients

This study was performed from September 2020 to September 2021. 85 patients were screened for eligibility, and 5 of them were excluded because of the exclusion criteria. Two patients had diagnosed with moderate to severe pulmonary insufficiency prior to the surgery. Three patients had trauma at the target acupoint stimulation site. Finally, eighty patients were enrolled and allocated randomly to the sham group (n = 40) or TEAS group (n = 40). A flow diagram of this study was shown in Fig. 1. Demographics and surgical information were balanced in two groups (*P* > 0.05) (Table 1).

**Table 1:** Demographic and Intraoperative Characteristics

	TEAS Group (n = 40)	Sham Group (n = 40)	*P*
Age (years)	56.15 ± 9.57	56.58 ± 9.53	0.832^△^
Gender (Male/Female)	15/25	19/21	0.366^&^
BMI (kg/m^2^)	24.86 ± 2.33	25.21 ± 2.50	0.445^△^
Smoking history (no/current)	34/6	35/5	0.745^&^
ASA rating (II/III)	33/7	28/12	0.189^&^
Comorbidities (n)			
Hypertension Coronary heart disease Diabetes	15(37.5%) 3(7.5%) 2(5%)	13(32.5%) 2(5%) 5(12.5%)	0.639^&^ 0.644^&^ 0.235^&^
Preoperative blood gas			
pH PaO_2_ (mmHg) PaCO_2_ (mmHg) Hb(g/L)	7.40 ± 0.12 88.08 ± 12.40 41.47 ± 3.07 137.13 ± 14.47	7.40 ± 0.02 84.25 ± 15.06 41.39 ± 2.90 137.13 ± 15.96	0.737^△^ 0.990^△^ 0.979^△^ 0.719^△^
Preoperative pulmonary function			
Preoperative FEV_1_ Preoperative FVC Preoperative FEV_1_/FVC	2.66 ± 0.46 3.29 ± 0.54 80.53 ± 3.52	2.78 ± 0.46 3.44 ± 0.57 80.72 ± 3.31	0.251^△^ 0.246^△^ 0.799^△^
Preoperative heart function			
LVEF (%)	64.75 ± 4.11	65.40 ± 4.74	0.385^△^
Type of surgery Lobectomy			
Lobectomy Wedge resection Segmentectomy	28(70.0%) 8(20.0%) 4(10.0%)	30(75%) 8(20.0%) 2(5.0%)	0.692^&^
Operative site			
Left/right	14/26	13/27	0.813^&^
Operation time (min)	140.75 ± 47.21	153.50 ± 53.32	0.261^△^
Anesthesia time (min)	182.88 ± 46.20	196.38 ± 52.78	0.227^△^
One lung ventilation time (min)	120.38 ± 45.64	133.25 ± 50.17	0.234^△^
Intraoperative fluid load (L)	1.32 ± 0.20	1.34 ± 0.16	0.642^△^
Intraoperative urine (L)	0.35 ± 0.03	0.35 ± 0.02	0.973^△^

Data are expressed as mean ± standard deviation or number of patients. ^△^Student’s *t*-test, ^&^Chi-square test. ASA: American Society of Anesthesiologists; BMI: body mass index (calculated as weight in kilograms divided by height in meters squared); FEV_1_: forced expiratory volume in one second; FVC: forced vital capacity; LVEF: left ventricle ejection fraction; TEAS: transcutaneous electrical acupoint stimulation.

### Changes of hemodynamics, blood gas, and lung function

No significant differences were identified in the values of HR, MAP, pH, Hb, and PaCO_2_ between both groups (*P* > 0.05). At T0, PaO_2_/FiO_2_, Qs/Qt, A-aDO_2_, and RI were comparable in two groups (*P* > 0.05). PaO_2_/FiO_2_ in two groups reduced obviously from T1 to T3 compared with T0 (*P* < 0.05). The levels of Qs/Qt, A-aDO_2_, and RI remarkably increased from T1 to T3 in two groups compared with T0 (*P* < 0.05). Compared with the sham group, TEAS significantly increased PaO_2_/FiO_2_ at T1 and T2 (*P* < 0.001). The levels of Qs/Qt, A-aDO_2_, and RI in the TEAS group obviously reduced from T1 to T3 (*P* < 0.05) (Table 2).

**Table 2:** Hemodynamics, blood gas, and lung function

	TEAS Group (n = 40)	Sham Group (n = 40)	*P*
HR (beats/min)			
T0 T1 T2 T3	76.03 ± 13.40 72.10 ± 9.34 70.43 ± 10.12 72.75 ± 11.13	74.00 ± 10.12 71.85 ± 8.76 68.97 ± 8.06 71.79 ± 9.68	0.448^△^ 0.902^△^ 0.481^△^ 0.741^△^
MAP (mmHg)			
T0 T1 T2 T3	99.33 ± 9.16 93.28 ± 5.67 94.10 ± 6.23 95.23 ± 6.50	98.38 ± 10.28 92.78 ± 7.35 92.80 ± 7.39 94.48 ± 7.81	0.664^△^ 0.734^△^ 0.398^△^ 0.642^△^
Hb (mg/L)			
T0 T1 T2 T3	135.51 ± 13.40 134.78 ± 12.61 134.28 ± 12.35 133.66 ± 11.71	136.03 ± 14.50 135.48 ± 13.90 135.08 ± 13.50 134.40 ± 12.87	0.868^△^ 0.815^△^ 0.784^△^ 0.787^△^
pH			
T0 T1 T2 T3	7.41 ± 0.04 7.40 ± 0.04 7.40 ± 0.04 7.38 ± 0.04	7.42 ± 0.04 7.41 ± 0.05 7.40 ± 0.05 7.38 ± 0.04	0.317^△^ 0.228^△^ 0.908^△^ 0.956^△^
PaCO_2_ (mmHg)			
T0 T1 T2 T3	40.58 ± 4.91 41.10 ± 5.35 40.23 ± 4.41 42.90 ± 4.42	40.79 ± 5.68 41.95 ± 5.51 41.83 ± 5.95 42.40 ± 5.12	0.862^△^ 0.486^△^ 0.176^△^ 0.643^△^
PaO_2_/FiO_2_ (mmHg)			
T0 T1 T2 T3	304.32 ± 52.42 180.43 ± 47.51^*#†^ 184.93 ± 33.93^*#†^ 245.13 ± 48.84^*†^	297.75 ± 66.89 105.15 ± 30.70^*†^ 128.18 ± 47.75^*†^ 221.53 ± 58.53^*†^	0.626^△^ 0.001^△^ < 0.001^△^ 0.056^△^
Qs/Qt (%)			
T0 T1 T2 T3	18.10 ± 2.23 22.94 ± 1.73^*#†^ 22.83 ± 1.25^*#†^ 20.39 ± 1.95^*#†^	18.34 ± 2.68 25.60 ± 1.09^*†^ 24.79 ± 1.70^*†^ 21.32 ± 2.19^*†^	0.341^△^ < 0.001^△^ < 0.001^△^ 0.026^△^
A-aDO_2_ (mmHg)			
T0 T1 T2 T3	357.95 ± 53.83 481.20 ± 46.80^*#†^ 477.79 ± 33.30^*#†^ 412.25 ± 49.77^*#†^	364.26 ± 65.63 555.41 ± 31.38^*†^ 532.54 ± 47.61^*†^ 438.47 ± 57.01^*†^	0.596^△^ < 0.001^△^ < 0.001^△^ 0.022^△^
RI			
T0 T1 T2 T3	1.25 ± 0.43 2.94 ± 1.11^*#†^ 2.69 ± 0.62^*#†^ 1.81 ± 0.62^*#†^	1.36 ± 0.63 5.78 ± 1.81^*†^ 4.90 ± 2.27^*†^ 2.24 ± 1.08^*†^	0.353^△^ < 0.001^△^ < 0.001^△^ 0.023^△^

Values expressed as mean ± standard deviation. ^*^P < 0.05 versus T0; ^#^*P* < 0.05 versus sham group. ^△^Student’s *t*-test, ^†^RM-ANOVA. HR: heart rate; MAP: mean arterial pressure; PaCO_2_: partial pressure of carbon dioxide partial pressure; A-aDO_2_: alveolar-arterial oxygen tension; RI: respiratory index; Qs/Qt: intrapulmonary shunt ratio; TEAS: transcutaneous electrical acupoint stimulation; RM-ANOVA: repeated measures analysis of variance. T0: before one-lung ventilation; T1: 30 min after one-lung ventilation; T2: 1 h after one-lung ventilation; T3: 10 min after resuming two-lung ventilation.

### Serum in MDA level and SOD activity

No significant differences were identified in MDA concentration and SOD activity in two groups at T0 and T1 (*P* > 0.05). Compared with T0, MDA levels increased in two groups from T1 to T3, SOD levels in both groups reduced at T2 and T3 (*P* < 0.05). At T2 and T3, the content of serum MDA was remarkably decreased, whereas SOD activity was remarkably increased compared with the sham group (*P* < 0.05) (Table 3).

**Table 3:** Changes in MDA level and SOD activity

	TEAS Group (n = 40)	Sham Group (n = 40)	*P*
MDA (nmol/mL)			
T0 T1 T2 T3	4.39 ± 0.63 4.94 ± 0.74^*†^ 5.27 ± 0.83^*#†^ 6.21 ± 0.89^*#†^	4.46 ± 0.61 5.01 ± 0.35^*†^ 5.66 ± 0.81^*†^ 7.10 ± 0.49^*†^	0.584^△^ 0.584^△^ 0.036^△^ < 0.001^△^
SOD (U/mL)			
T0 T1 T2 T3	463.26 ± 26.98 458.01 ± 18.86 423.80 ± 16.85^*#†^ 405.79 ± 27.25^*#†^	464.51 ± 30.90 457.76 ± 22.19 412.80 ± 13.50^*†^ 387.22 ± 24.35^*†^	0.848^△^ 0.957^△^ 0.002^△^ 0.002^△^

Data are expressed as mean ± standard deviation. ^*^P < 0.05 versus T0; ^#^*P* < 0.05 versus sham group.^△^Student’s *t*-test, ^†^RM-ANOVA. MDA: malondialdehyde; SOD: superoxide dismutase; TEAS: transcutaneous electrical acupoint stimulation; RM-ANOVA: repeated measures analysis of variance. T0: before one-lung ventilation; T1: 30 min after one-lung ventilation; T2: 1 h after one-lung ventilation; T3: 10 min after resuming two-lung ventilation.

### Other clinical indicators

No significant differences were identified in the removal time of the thoracic drainage tube, the incidence of pleural effusion and pulmonary atelectasis between both groups (*P* > 0.05). The duration of ICU stay and hospital stay after surgery in the TEAS group was obviously shortened (*P* < 0.05). Compared with the sham group, the QoR-15 score at postoperative day1 and day2 was significantly higher in TEAS group (*P* < 0.05) (Table 4).

**Table 4:** Other clinical endpoints

	TEAS Group (n = 40)	Sham Group (n = 40)	*P*
Pleural effusion	11(27.5%)	13(32.5%)	0.626^&^
Atelectasis	2(5%)	7(17.5%)	0.157^&^
The duration of ICU stay (hours)	21.03 ± 6.92^*^	27.93 ± 12.42	0.003^△^
Thoracic drainage tube removal time (days)	3.44 ± 1.31	3.29 ± 1.21	0.597^△^
The length of postoperative hospitalization (days)	5.76 ± 1.35^*^	6.45 ± 1.66	0.046^△^
QoR-15 on postoperative day 1	114.33 ± 4.55^*^	111.70 ± 3.00	0.003^△^
QoR-15 on postoperative day 2	130.05 ± 3.88^*^	126.85 ± 4.25	0.001^△^

Data are expressed as mean ± standard deviation or number of patients. ^*^P < 0.05 versus sham group. ^△^Student’s *t*-test, ^&^Chi-square test. ICU: intensive care unit; QoR-15: Quality of Recovery-15; TEAS: transcutaneous electrical acupoint stimulation.

### Adverse events

Potential adverse events of TEAS include continuous post-electrostimulation sensation and skin numbness. All adverse reactions will be recorded. If acute or other serious adverse reactions occur, the participants will be asked to discontinue from this trial and accepted medical attention immediately. Serious adverse events will be reported to the ethics committee. There were no adverse events occurred in our research.

## Discussion

This randomized controlled trial demonstrated that TEAS could inhibit oxidative stress during OLV in patients with lung cancer, thereby improving oxygenation index and pulmonary diffusion function. Additionally, TEAS could shorten the postoperative ICU and hospital stay, and improve the QoR-15 score. These results revealed that the application of TEAS during OLV may benefit patients with lung cancer undergoing thoracic surgery.

One-lung ventilation is a common ventilation method used in thoracic anesthesia for airway management, which could not only prevent cross-infection and metastasis of secretions and blood from the operated side to the healthy side lung but also create a clear surgical field. However, during lung resection and OLV, lots of factors, including ventilator-induced lung injury (VILI), hypoxic pulmonary vasoconstriction (HPV), ischemia-reperfusion injury, and surgical trauma, could lead to pulmonary mechanical and biochemical injuries. Up to date, a large number of studies had shown that oxidative stress was one of the key potential mechanisms underlying lung injury during OLV in thoracic surgery [19, 20]. Therefore, it is of great importance to reduce oxidative stress, thereby reducing postoperative pulmonary complications and improving prognosis.

As an innovative type of acupuncture therapy, TEAS transmitted a specific low-frequency pulse current to the body through electrodes, which had a similar effect to electroacupuncture. Previous research suggested that electroacupuncture may reverse pulmonary inflammation and oxidative damage in rats by reducing p38 phosphorylation along with caspase-3 activation [21]. Compared with electroacupuncture, TEAS induced less pain and injury and had a lower incidence of infection. Many studies had suggested that stimulation of certain acupuncture points could significantly improve lung function and patients’ quality of life [22–24]. Feishu (BL13) is a classic point for improving lung function. Acupuncture treatment of BL13 can be used to treat respiratory diseases such as cough, asthma, and chest tightness [25, 26]. Because the T1-T5 nerve segments dominate the lungs and BL13 is associated with T3. As previously reported, electroacupuncture stimulation of ST16 and BL13 can reduce the postoperative inflammatory response in elderly patients undergoing radical resection of lung cancer, thus decreasing postoperative pulmonary complications [27]. A research indicated that electroacupuncture stimulation at ST16 and BL13 can reduce lung injury induced by limb ischemia- reperfusion through inhibiting pro-inflammatory cytokine response and oxidative stress [28]. Another study revealed that electroacupuncture pretreatment at Hegu (LI4) could alleviate LPS-induced acute respiratory distress syndrome via regulating the PPARγ/NF-kB signaling pathway [29]. Therefore, this study chose BL13, LI4, and ST36 as target acupoints.

During one-lung ventilation, non-ventilated lung not only collapsed in alveoli but also decreased blood perfusion by 50% due to HPV and gravity, which directly led to vascular structure destruction and increased ROS production [30, 31]. Previous study has shown that inhibition of ROS production favors HPV, but decreased SOD activity aggravates oxidative stress and then weakens the effect of HPV [32]. Moreover, peripheral blood leukocytes and activated endogenous microglia increase the formation of oxygen free radicals during ischemia-reperfusion injury and cause necrosis of cell tissues, which leads to a cascade of inflammatory responses and promotes oversecretion of inflammatory cytokines, such as IL-1, IL-6, IL-8 and TNF-α [33, 34]. Therefore, oxidative stress and inflammatory reactions might damage normal tissue. The cell damage after hypoxia is biphasic, which begins with hypoxia and aggravates during reoxygenation. Study had shown that the production of large amounts of free radicals led to reoxygenation lung injury after thoracotomy [35]. Consistent with other researches, we found that serum MDA content was remarkably increased and antioxidant enzyme SOD activity significantly decreased during one-lung ventilation compared to baseline, as well as increased shunt fraction, which suggested that oxidative stress may weaken the protective effect of HPV [32, 36]. Furthermore, previous study demonstrated that the levels of 8-iso-PGF2α, nitrites plus nitrates and hydrogen peroxide in blood and exhaled breath condensate increased during one-lung ventilation [37], which provided further evidence for oxidative lung injury in patients during lobectomy. In this study, we found that MDA level was obviously reduced and SOD activity was significantly increased in the TEAS group, indicating that TEAS can regulate oxidative stress undergoing thoracic surgery.

PaO_2_/FiO_2_ is commonly used to detect pulmonary gas exchange and oxygenation function, which also is the main diagnostic indicator of ARDS. OLV could cause functional shunt of ventilation-perfusion imbalance, resulting in increased Qs/Qt and decreased PaO_2_ [38]. Moreover, A-aDO_2_ and RI could reflect the pulmonary diffusion function, and positively correlate with the degree of lung damage [39]. Therefore, the protective effect of TEAS on lung function was evaluated by PaO_2_/FiO_2_, Qs/Qt, A-aDO_2_, and RI in present study. Studies had shown that the ratio of ventilation volume to blood flow was reduced to some extent due to lung cancer resection, thus leading to hypoxemia and pulmonary function damage [39, 40]. In our study, it was also observed that the levels of Qs/Qt, A-aDO_2_, and RI in both groups increased during one-lung ventilation, whereas the PaO_2_/FiO_2_ decreased, which indicated that OLV can impair lung function. Nevertheless, PaO_2_/FiO_2_ was significantly improved in the TEAS group at T1 and T2, while Qs/Qt, A-aDO_2_, and RI considerably decreased from T1 to T3, which proposed that TEAS could reduce the imbalance of ventilation/perfusion, and improve lung diffusion and oxygenation capacity. Studies revealed that TEAS could improve oxygenation by increasing PaO_2_ and decreasing A-aDO_2_ in patients during OLV [41, 42], which were in support of our study. Researches also found that the improvement of lung function was closely related to the regulation of oxidative damage [43, 44]. Moreover, Pen et al. suggested that electroacupuncture may improve local blood circulation by regulating the balance of endothelium-derived vasoconstrictors and vasodilators [45]. Therefore, combined with the above results, our findings observed that inhibition of oxidative stress may be important mechanisms for TEAS to improve pulmonary function during one-lung ventilation in patients with lung cancer.

Additionally, the incidence of pleural effusion and atelectasis was compared between the two groups. However, the duration of ICU stay and hospital stay after surgery was markedly reduced in the TEAS group, possibly due to reduced oxidative stress [46]. The QoR-15 score can effectively evaluate the quality of postoperative recovery of patients, which had been widely used in clinical trials and studies. In our study, the QoR-15 score at postoperative day 1 and 2 was significantly increased in the TEAS group, indicating that the quality of postoperative recovery was improved.

There are some limitations in this study. The main limitation of our research was the relatively small study population. Considering the inherent bias of a small number of samples, the results of our study need to be confirmed by further research. Also, further research was needed to explore other possible protective mechanisms associated with TEAS.

## Conclusions

To sum up, we consider that the application of TEAS could attenuate oxidative lung injury during OLV, thereby protecting the pulmonary function and effectively promoting the early recovery of patients with lung cancer.

## Data Availability

The datasets used and analyzed in this study are available on reasonable request from the corresponding author (hblijianli@163.com).

## Abbreviations

ALI: Acute lung injury
ARDS: Acute respiratory distress syndrome
ASA: American Society of Anesthesiologists
BIS: Bispectral index
DLT: Double-Lumen Tube
ELISA: Enzyme linked immunosorbent assay
FEV1: Forced expiratory volume in 1 s
FiO_2_: Fraction of inspired oxygen
HR: Heart rate
ICU: Intensive care unit
MDA: Malondialdehyde
HPV: Hypoxic pulmonary vasoconstriction
MAP: Mean arterial pressure
OLV: One-lung ventilation
PACU: Postanesthesia care unit
PCV-VG: Pressure-controlled ventilation-volume guaranteed
PEEP: Positive end-expiratory pressure
QoR-15: Quality of Recovery-15
Qs/Qt: Intrapulmonary shunt ratio
RI: Respiratory index
RM-ANOVA: Repeated measures analysis of variance
ROS: Reactive oxygen species
SOD: Superoxide dismutase
SpO_2_: Peripheral capillary oxygen saturation
TEAS: Transcutaneous electrical acupoint stimulation
TENS: Transcutaneous electrical nerve stimulation
VILI: Ventilator-induced lung injury
